# Supplementary material for: Association between oral microbiome and seven types of cancers in East Asian population: a two-sample Mendelian randomization analysis
Source: Front Mol Biosci. 2023 Nov 21;10:1327893. doi: 10.3389/fmolb.2023.1327893 (PMC10702768; doi:10.3389/fmolb.2023.1327893)
Supplement: Supplementary file 1 [file DataSheet1.ZIP › Supplementary Materials/MR plots of saliva/saliva═╝/Gastric cancer/pheno.3363_to_gastric cancer_leave_one_out.pdf]

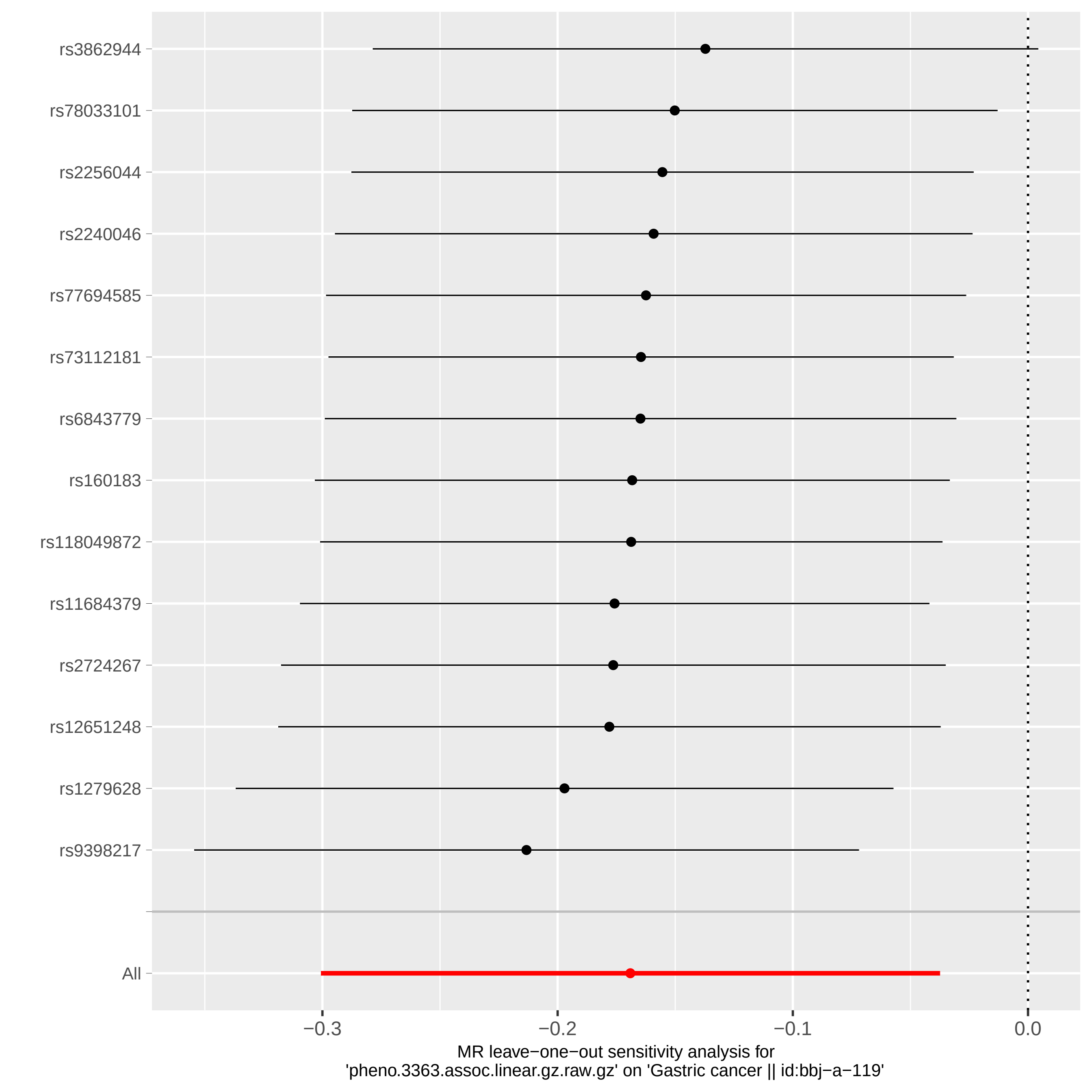

rs3862944
rs78033101
rs2256044
rs2240046
rs77694585
rs73112181
rs6843779
rs160183
rs118049872
rs11684379
rs2724267
rs12651248
rs1279628
rs9398217
All
−0.3
−0.2
−0.1
0.0
MR leave−one−out sensitivity analysis for
'pheno.3363.assoc.linear.gz.raw.gz' on 'Gastric cancer || id:bbj−a−119'
